# Effect and Prognosis Factors of Combining Laparoscopic Radical Resection of Colon Adenocarcinoma with Docetaxel Therapy in Treating Middle and Advanced Colon Adenocarcinoma

**DOI:** 10.1155/2022/6122261

**Published:** 2022-05-27

**Authors:** Qi Gao, Caifeng Zhang, Zhichao Dong, Yan Guo, Li Zhang

**Affiliations:** ^1^Department of Gastroenterology, Xinxiang Central Hospital, The Fourth Clinical College of Xinxiang Medical College, Xinxiang 453000, Henan, China; ^2^Department of Gastroenterology, The First Affiliated Hospital of Xinxiang Medical College, Xinxiang 453100, Henan, China

## Abstract

**Objective:**

The aim of the study is to explore the clinical efficacy and prognosis factors of joint application of laparoscopic radical resection of colon adenocarcinoma (COAD) and docetaxel therapy in treating COAD of middle and advanced stages.

**Methods:**

The clinical data of 103 COAD patients of middle and advanced stages treated in our hospital from July 2016 to July 2018 were selected for the retrospective analysis, all patients received the treatment scheme of combining laparoscopic radical resection of COAD with docetaxel therapy for the observation of short-term efficacy, follow-up was conducted to record their 3-year survival, and relevant factors affecting patient prognosis were analyzed by the logistic regression model.

**Results:**

After treatment, the total remission rate of patients was 75.73% (78/103), the total incidence rate of adverse reactions was 16.50% (17/103); patients' level values of various serum tumor markers after treatment were significantly lower than those before treatment (*P* < 0.001); according to the univariate analysis results, for COAD patients with different tumor diameters, differentiated degrees, TNM stages, perineural invasion degrees, pathological types, and depths of invasion, their modality rates were statistically different (*P* < 0.05); and the logistic regression analysis showed that tumor diameter ≥5 cm, poor differentiation, TNM stage IV, perineural invasion, pathologically signet-ring cell carcinoma, and *T*_3_-invasion were the independent risk factors affecting patient prognosis (*P* < 0.05).

**Conclusion:**

Combining laparoscopic radical resection of COAD with docetaxel therapy in treating COAD of middle and advanced stages achieves affirmed short-term efficacy, which can reduce patients' level of serum tumor markers and ensure high safety and good survival prognosis. Tumor diameter, differentiated degree, TNM stage, perineural invasion, pathological type, and *T*_3_-invasion are the relevant factors affecting the prognosis of middle and advanced COAD.

## 1. Introduction

Colon adenocarcinoma (COAD) is relatively common among the malignant neoplastic diseases in the digestive system [1]; its incidence varies greatly around the world, which is exceedingly high in North America and Europe. In recent years, with the high rate of economic development in China, gradual improvement of people's living standards, and the “Westernization” of the dietary structure, China's incidence and mortality of COAD are getting close to regions such as North America and Europe, making COAD the third most malignant tumor disease in China [2]. COAD is usually treated by surgery, mostly by traditional laparotomy, to remove the lesion completely, but the long surgery time, large intraoperative blood loss, great trauma, etc. can cause damage to the patients' body and poor prognosis [3]. Laparoscopic surgery, on the other hand, enables precise ligation of tumor vessels, which can achieve the same degree of tumor resection compared to traditional laparotomy. However, it is difficult for the two surgery modalities to reach a desirable treatment effect, and patients need to accept an adjuvant drug therapy. Docetaxel is a new generation of taxel antitumor drugs that can control disease progression by enhancing tubulin polymerization and inhibiting microtubule depolymerization to disrupt mitosis of tumor cells, and arresting tumor cell growth [4], which has been applied in treating breast cancer, cervical cancer, and ovarian cancer [5–7]. COAD prognosis is generally associated with differentiation degree, tumor stage and pathological type, but because of the influence of other factors, it is still quite different even in the same condition. Therefore, the prognosis can be affected by multiple complex factors, and the related indicators need to be continuously improved to guide the treatment of the disease [8]. At present, the treatment of COAD is mostly based on surgery and supplemented by postoperative chemotherapy, and the evaluation of postoperative prognosis and the development of an intervention program can effectively improve the long-term outcome of patients. Based on this, the clinical efficacy of laparoscopic radical resection of COAD combined with docetaxel therapy in treating COAD of middle and advanced stages was analyzed and the factors affecting patient outcome were explored in the study, so as to guide future clinical intervention and then improve prognosis.

## 2. Materials and Methods

### 2.1. General Data

The study subjects were 103 patients with middle and advanced COAD treated in our hospital from July 2016 to July 2018, and the study met the criteria of World Medical Association Declaration of Helsinki (2013) [9]. Inclusion criteria were as follows: (1) those who were diagnosed with COAD for the first time by colonoscopy and met the relevant diagnosis criteria for COAD in *Clinical Diagnosis and Treatment Guide · Tumor Staging*; (2) those who met the surgical indications of laparoscopic surgery, and their estimated survival was at least 6 months; and (3) those who did not have chemotherapy contraindications, and received the docetaxel therapy for the first time. Exclusion criteria were as follows: (1) those who obtained abnormal results in electrocardiogram, routine examination of blood, urine, and stool, and liver and kidney function tests; (2) those who were complicated with neurologic disorder history, diabetic peripheral neuropathy, and autoimmune diseases; and (3) those who were allergic to the drug used in the study or quit the trial in the middle of treatment. The study was approved by the Hospital Ethics Committee.

### 2.2. Methods

The patients received laparoscopic radical resection of COAD under general anesthesia. With the patients in the Trendelenburg position, an incision was made at the navel site for establishment of CO_2_ pneumoperitoneum to infuse CO_2_ at the pressure controlled within 12–14 mmHg. Three to four puncture holes were made into the lesion at the left and right upper abdomen to place the 5 mm trocars, the 10 mm trocars were placed through the lower part of umbilicus and both sides of upper abdomen for placement of ultrasound knife, forceps and laparoscope for intra-abdominal exploration and observation of tumor size, position, and metastases. After the tumor location was determined, the blood vessel was ligated at the root, the local intestinal canal and mesenterium proximal to the tumor were tied with gauze, the peritoneum and mesenterium were cut open with ultrasound knife to free the part from mesenterium vessel to the root, resect the sigmoid colon and the lymph glands under the mesenterium at both sides of the rectum from top to bottom, remove the excess fat tissue, and cut open the colic ligament and half of the gastrocolic ligament. An anastomat was placed in the anus of patients to anastomose with the resected intestinal canal. After the end of surgery, the patients' abdominal cavity was rinsed with normal saline and a discharge tube was placed.

After surgery, docetaxel (manufacturer: Jiangsu Hengrui Medicine Co. Ltd.; registration number: X20020450; specification: 60 mg) was given via intravenous drip.

### 2.3. Observation Indicators

#### 2.3.1. Evaluation of Short-Term Efficacy

According to the Response Evaluation Criteria in Solid Tumors (RECIST) [10], the patients' treatment effect was evaluated (see Table 1 for the specific criteria), which was classified as complete response (CR), partial response (PR), stable disease (SD), and progressive disease (PD). Moreover, the total remission rate = (number of CR cases + number of PR cases)/total number of cases × 100%.

#### 2.3.2. Survival

The patients' 3-year overall survival (OS) was recorded, and their clinical and pathological files were collected and organized for the analysis of factors affecting prognosis. Distant metastasis: metastases of cancer foci to the organs or lymph glands other than the surgical site were observed.

#### 2.3.3. Detection of the Serum Tumor Marker Level

The level values of serum CEA, CA125, and CA19-9 were measured by the Cobas E411 analyzer that applies the electrochemiluminescence (ECL) technology (manufacturer: Guangzhou Golon Technology Co., Ltd.).

### 2.4. Follow-Up

All patients were followed up every 3 months by outpatient review, telephone, and other modalities, the review contents included imaging examinations and oncological indicators, and enteroscopy was rechecked every year to track the patients' tumor metastasis and survival.

### 2.5. Statistical Methods

In this study, the data were processed by the professional statistical software SPSS26.0, the picture drawing software was GraphPad Prism 7 (GraphPad Software, San Diego, USA), the enumeration data were examined by *X*^2^ test and expressed by (n(%)), the measurement data were examined by *t*-test and expressed by mean ± SD, the risk factors affecting the prognosis of patients with middle and advanced COAD were analyzed by logistic regression, and differences were considered statistically significant at *P* < 0.05.

## 3. Results

### 3.1. Efficacy Evaluation

After treatment, the patients' clinical symptoms were improved, and there were 36 CR cases (34.95%), 42 PR cases (40.78%), 16 SD cases (15.53%), and 9 PD cases (8.74%). The total remission rate was 75.73% (78/103).

### 3.2. Occurrence of Complications

During the study, 4 patients had postoperative bleeding (3.88%), 3 patients had myelosuppression (2.91%), 4 patients had cutaneous toxic reactions (3.88%), and 6 patients had mild thrombocytopenia (5.83%). The total incidence rate of adverse reactions was 16.50% (17/103).

### 3.3. Survival and Distant Metastasis

According to the 3-year follow-up to 76 patients, the distant metastasis rate was 23.30% (24/103); and the 3-year OS was 75.73% (78/103).

### 3.4. Comparison of Serum Tumor Marker Levels before and after Treatment

**Figure 1:**
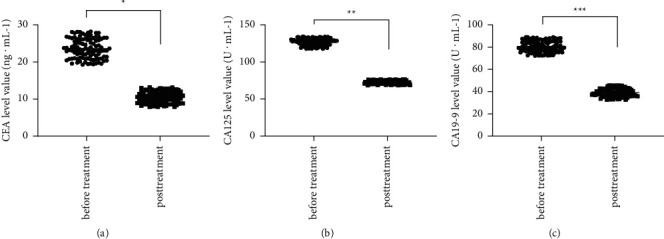
Comparison of patients' serum tumor marker levels before and after treatment (mean ± SD). (a) the comparison of serum CEA level values of patients with middle and advanced COAD before and after treatment, the horizontal axis indicated before treatment and after treatment, and the vertical axis indicated the CEA level value (ng·mL^−1^); before and after treatment, the mean serum CEA level values of patients with middle and advanced COAD were, respectively, (23.62 ± 2.65) and (10.42 ± 1.51); ^*∗*^significant difference in the mean serum CEA level values of patients with middle and advanced COAD before and after treatment (*t* = 43.923, *P* < 0.001); (b) the comparison of serum CA125 level values of patients with middle and advanced COAD before and after treatment, the horizontal axis indicated before treatment and after treatment, and the vertical axis indicated the CA125 level value (U·mL^−1^); before and after treatment, the mean serum CA125 level values of patients with middle and advanced COAD were, respectively, (127.05 ± 4.68) and (72.72 ± 2.36); ^*∗∗*^significant difference in the mean serum CA125 level values of patients with middle and advanced COAD before and after treatment (*t* = 105.199, *P* < 0.001); (c) the comparison of serum CA19-9 level values of patients with middle and advanced COAD before and after treatment, the horizontal axis indicated before treatment and after treatment, and the vertical axis indicated the serum CA19-9 level value (U·mL^−1^); before and after treatment, the mean serum CA19-9 level values of patients with middle and advanced COAD were, respectively, (80.67 ± 5.03) and (39.24 ± 3.65); ^*∗∗∗*^significant difference in the mean serum CA19-9 level values of patients with middle and advanced COAD before and after treatment (*t* = 67.656, *P* < 0.001).

After treatment, the patients' level values of various serum tumor markers were significantly lower than those before treatment (*P* < 0.001). See Figure 1.

### 3.5. Univariate Analysis of Factors Affecting Patient Survival

The differences in mortality rates of COAD patients with different gender and tumor location were not statistically significant (*P* > 0.05); and for COAD patients with different tumor diameters, differentiated degrees, TNM stages, perineural invasion, pathological types, and depths of invasion, their mortality rates were statistically different (*P* < 0.05). See Table 2.

### 3.6. Multivariate Analysis of Factors Affecting Patient Survival

The logistic regression analysis showed that tumor diameter≥5 cm, poor differentiation, TNM stage IV, perineural invasion, pathologically signet-ring cell carcinoma, and *T*_3_-invasion were the independent risk factors affecting patient prognosis (*P* < 0.05). See Table 3.

## 4. Discussion

The latest report showed [11] that COAD incidence and mortality respectively rank 3^rd^ and 4^th^ in all malignant tumors, of which the incidence rose from 5^th^ to 3^rd^ in three years (2015 to 2018) according to the relevant study data, demonstrating that the incidence of COAD in China is increasing on a yearly basis. Surgical resection is still the main treatment of COAD [12], and the short-term efficacy of laparoscopic radical resection for COAD has been recognized. Clinical studies found that [13, 14] performing laparoscopic radical resection to COAD patients who present with surgical indications can achieve the same results as an open surgery. Since the 1990s, the laparoscopic technology has been continuously improved, and with the help of laparoscope, physicians can clearly recognize the target region anatomy and intuitively identify the lesion condition, peripheral adhesion manifestations, etc., and then accurately perform tissue separation, greatly reducing the interference with peripheral tissues [15, 16]. During the surgical procedure, by connecting the laparoscope with TV amplification system, the dissection along the perirectal space can be accurately performed, and the deep layer of the tumor can be separated by using the ultrasound knife for sharp dissection and following the “En bloc resection” principle [14]. When removing the foci by using an ultrasound knife, effective hemostasis through the tissue such as the abdominal cavity and mesenterium minimizes the intraoperative blood loss, and with the assistance of laparoscope, the lymph nodes can be completely removed, thereby reducing the surgical incision and the risk of lesion metastasis and improving treatment efficacy and safety [17, 18].

COAD is one of the major diseases that endangers human health, and although surgical resection combined with postoperative adjuvant chemotherapy is the standard treatment for COAD in middle and advanced stages, some patients who receive the regimen still experience recurrence and metastasis, leading to shorter survival [19]. In recent years, COAD treatment has achieved a tremendous progress with the development and clinical application of molecular targeted agents such as cetuximab and bevacizumab as well as immunotherapeutic agents, but currently, there is a lack of evidence for their effectiveness in the postoperative adjuvant treatment of COAD, and how to effectively alleviate the toxic effects of postoperative adjuvant chemotherapy in patients, reduce the recurrence rate, and prolong the survival remains an important issue to be solved currently. Docetaxel is a new antitumor drug with a unique anticancer mechanism, and its precursor is obtained by semisynthesis of the extract from the needle of European yew. Docetaxel can promote tubulin polymerization, inhibit tubulin depolymerization, and thus form a stable nonfunctional microtubule and suppress tumor cell proliferation and division, with an activity that is 11.3-12 times that of taxol and effects that have been demonstrated in advanced gastric cancer, lung cancer, nasopharyngeal cancer, and other diseases [20]. The trial further confirmed the efficacy of combining laparoscopic radical resection of COAD with docetaxel therapy in patients with middle and advanced COAD, so as to provide more basis for improving such patients' quality of survival and prolonging their survival.

In this study, 103 patients with middle and advanced COAD treated in our hospital were collected as the subjects, and the joint application of laparoscopic radical resection of COAD and docetaxel therapy was conducted to explore the clinical effect of the treatment plan. CEA is a common tumor marker closely related to the occurrence of malignant tumors, which can be used for the diagnosis, treatment, and prognosis evaluation of COAD. CA19-9 is a common gastrointestinal tumor indicator, which has a good value in the prognosis evaluation and efficacy judgment of COAD. CA125 is used clinically as a tumor marker for ovarian cancer and is important in the diagnosis and monitoring of the extent of progression and recurrence status of ovarian cancer [21], which was subsequently found to have a high positive detection rate in malignant cancer diseases such as gastric cancer, COAD, and pancreatic cancer [22]. In the study by some scholars [23], Xiaoaiping tablets combined with docetaxel and cisplatin therapy was given to patients with middle and advanced COAD, and the serum CEA level values of patients before and after treatment were, respectively, (33.76 ± 4.24) ng/mL and (18.44 ± 2.56) ng/mL; while in this study, the patients' serum CEA level after treatment was significantly better than the above experimental result, indicating that the combined treatment can more effectively kill the COAD cells and inhibit disease progression. In this study, logistic regression analysis was used to explore the effect of multiple factors on disease prognosis and provide a clinical basis for improving patients' quality of survival. The study found that tumor diameter ≥5 cm, poor differentiation, TNM stage IV, perineural invasion, pathologically signet-ring cell carcinoma, and *T*_3_-invasion were the independent risk factors affecting patient prognosis. In patients with a high TNM stage, the invasion of tumor tissues into the intestinal wall is deep, the progression rate and degree of malignancy are high, and the invasiveness is strong, so cancer cells are more likely to spread and metastasize, leading to poor prognosis of patients. In addition, tissue differentiation can have some effects on tumor biological behavior, poorly differentiated tumors have strong regeneration ability, and cancer cells grow fast and are more prone to lymph node metastasis, producing malignant biological behavior and affecting prognosis. Moreover, for patients with an infiltration depth of *T*_3_, the tumor tissue is difficult to be removed completely, and once the residual tumor tissue penetrates the muscularis into the serosa, the lymph node and hematogenous dissemination will be accelerated, causing tumor invasion and metastasis as well as poor prognosis [24]. Although laparoscopic treatment has a good therapeutic effect, it requires the operators' good grip of knowledge of dissection and abdominal cavity, a long learning curve, and a higher treatment cost due to the special characteristics of this technique; for patients with large tumor volume, the space around the lesion is relatively small, which affects the surgical field to some extent and is not conducive to the treatment. This study was limited by many factors, such as fewer indicators and lack of mass data on the efficacy and safety of disease treatment, so the trial design should be refined in future studies to provide more reference basis for the clinical treatment of COAD patients.

Combining laparoscopic radical resection of COAD with docetaxel therapy provides an important basis for prolonging the survival of patients with middle and advanced COAD and improving the clinical efficacy, which is a great advance and development of COAD treatment and is extensively instructive in the clinical treatment of tumor diseases. Such modality undoubtedly provides a new direction in the treatment of COAD.

## Figures and Tables

**Table 1:** RECIST criteria.

Efficacy	Criteria
CR	Disappearance of all lesions for at least 4 weeks
PR	Lesion with dual measurable diameters, >50% decrease in the sum of the longest diameters (SLD) of the targeted lesions for at least 4 weeks
SD	Lesion with dual measurable diameters, neither sufficient decrease to qualify for PR nor sufficient increase to qualify for PD, for at least 4 weeks
PD	>25% increase of one or more lesions, or appearance of new lesions

**Table 2:** Univariate analysis of factors affecting patient survival.

Factor	Cases	Mortality (*n*(%))	*X* ^2^	*P*
Gender			0.006	0.939
Male	57	14 (24.56)		
Female	46	11 (23.91)		
Tumor diameter (cm)			12.253	<0.001
<5	67	9 (13.43)		
≥5	36	16 (44.44)		
Tumor location			0.281	0.501
Ascending colon	19	5 (26.32)		
Transverse colon	23	7 (30.43)		
Descending colon	25	5 (20.00)		
Sigmoid colon	36	8 (22.22)		
Differentiated degree			5.373	<0.05
Poor	36	16 (44.44)		
Moderate	51	7 (13.73)		
Well	16	2 (12.50)		
TNM stage			21.271	<0.001
III	68	7 (10.29)		
IV	35	18 (51.43)		
Perineural invasion			9.582	<0.05
Yes	32	14 (43.75)		
No	71	11 (15.49)		
Pathological type			5.373	<0.05
Signet-ring cell carcinoma	36	16 (44.44)		
Tubular adenocarcinoma	16	2 (12.50)		
Mucinous adenocarcinoma	51	7 (13.73)		
Depth of invasion			5.373	<0.05
*T*_1_	16	2 (12.50)		
*T*_2_	51	7 (13.73)		
*T*_3_	36	16 (44.44)		

**Table 3:** Multivariate analysis of factors affecting patient survival.

Factor	B	SE	Wald	*P* value	Exp	95%CI
Tumor diameter ≥5 cm	0.430	0.462	3.867	0.034	2.743	1.032–5.382
Poor differentiation	1.127	0.361	9.823	0.025	3.162	1.162–4.382
TNM stage IV	1.352	0.473	6.732	0.008	1.435	1.028–1.938
Perineural invasion	0.263	0.683	4.281	0.013	3.271	1.198–5.372
Pathologically signet-ring cell carcinoma	1.024	0.296	4.082	0.004	2.374	1.034–3.846
*T*_3_-invasion	1.362	0.463	11.263	0.025	3.273	1.123–4.372

## Data Availability

The data used to support the findings of this study are available on reasonable request from the corresponding author.
